# Metabolic (dysfunction)-associated fatty liver disease metrics and contributions to liver research

**DOI:** 10.1007/s12072-024-10731-0

**Published:** 2024-10-16

**Authors:** Maito Suoh, Saeed Esmaili, Mohammed Eslam, Jacob George

**Affiliations:** Storr Liver Centre, The Westmead Institute for Medical Research, Westmead Hospital and The University of Sydney, 176 Hawkesbury Rd, Westmead, NSW 2145 Australia

**Keywords:** MAFLD, Cardiovascular disease, Chronic kidney disease, Liver fibrosis, COVID-19, Bibliometrics, Bibliometric analysis, Bibliographic network, Co-occurrence network, Co-citation network

## Abstract

**Background:**

The international consensus to revise non-alcoholic fatty liver disease to metabolic (dysfunction)-associated fatty liver disease (MAFLD) in 2020 attracted significant attention. The impact of the MAFLD definition on the research community has not been objectively assessed. We conducted an analysis of systematically collected literature on MAFLD to understand its research impact.

**Methods:**

From PubMed, Web of Science, and Scopus, the literature adopting MAFLD, written in English, and published from 2020 to 10 October 2023 was collected. The publication metrics, including publication counts, publishing journals, author countries, author keywords, and citation information, were analyzed to evaluate the research impact and key topics on MAFLD.

**Results:**

1469 MAFLD-related papers were published in 434 journals with a steady increase in the number. The intense publishing and citations activity on MAFLD indicates the large impact of the redefinition. Topic assessment with keyword and citation analysis revealed a transition from the proposal and discussion of the redefinition to clinical characterization of MAFLD with a focus on metabolic dysfunction. Moreover, the diagnostic criteria for MAFLD showed better performance in predicting hepatic and extrahepatic outcomes compared to NAFLD. The publications were from 99 countries with evidence of strong regional and global collaboration. Multiple international societies and stakeholders have endorsed MAFLD for its utility in clinical practice, improving patient management and promoting multidisciplinary care, while alleviating stigma.

**Conclusion:**

This survey provides a quantitative measure of the considerable international impact and contributions of the MAFLD definition towards liver research and as part of the spectrum of cardiometabolic disorders.

**Supplementary Information:**

The online version contains supplementary material available at 10.1007/s12072-024-10731-0.

## Introduction

The burden of fatty liver disease is now a major cause of adverse hepatic outcomes including cirrhosis and liver cancer [1]. Moreover, this disease in context is more broadly the liver manifestation of systemic metabolic dysfunction that is closely aligned to adverse cardiometabolic outcomes, particularly cardiovascular disease (CVD) and chronic kidney disease (CKD) [1–5]. Despite this knowledge, fatty liver disease associated with metabolic dysfunction was termed non-alcoholic fatty liver disease (NAFLD) and diagnosed primarily by the exclusion of other liver disease etiologies, particularly excess alcohol intake [2, 3]. To emphasize the link to pathogenesis, an international consensus panel proposed the term metabolic (dysfunction)-associated fatty liver disease (MAFLD) and a set of diagnostic criteria in 2020 [2, 3]. The diagnosis of MAFLD is based on the presence of metabolic dysregulation, including obesity and type 2 diabetes mellitus, regardless of concomitant liver disease [2, 3]. The overarching goal of the redefinition is to improve clinical practice and research by reframing our understanding of fatty liver disease [2].

The introduction of MAFLD as a term led to numerous consequences. It not only incited intense discussion [6–9], but also attracted endorsements from multiple organizations and stakeholders [1, 10–13]. Furthermore, updating the diagnostic criteria for fatty liver disease resulted in many studies investigating the clinicopathological features and diagnostic validity of MAFLD [14]. The MAFLD proposal coincided with the pandemic from coronavirus disease 2019 (COVID-19) [15, 16]. Consequently, MAFLD was also examined as a risk factor for severe COVID-19, together with other liver diseases [15] or other metabolic conditions such as obesity and type 2 diabetes [16]. Although many of these topics have been extensively described elsewhere, the overall impact of MAFLD on research activity has not been objectively and comprehensively assessed.

In 2023, other pan-national societies proposed metabolic dysfunction-associated steatotic liver disease (MASLD) to replace NAFLD [17]. As compared to MAFLD, MASLD requires the exclusion of concomitant liver diseases as in NAFLD, and the requirement for metabolic factors also differs [17]. In this rapidly evolving landscape where two different terms and criteria are proposed to replace NAFLD, it is important to objectively and critically assess the research status of MAFLD.

Bibliometric studies aim to make inferences from the statistical analysis of a large body of literature within a specific topic that is unsuitable for manual review [18]. Although several bibliometric studies exist in this field, these analyzed NAFLD alone [19] or NAFLD and MAFLD altogether [20–23], making it difficult to evaluate the specific contributions with respect to MAFLD. Hence, to quantitatively and systematically evaluate the research impact and the consequences related to MAFLD, we conducted a survey of publication metrics or bibliometrics in the literature adopting the term MAFLD. In this study, publication counts, publishing journals, and authoring countries were analyzed to quantify research activity, whereas author keywords and citation information were used to identify key research topics and findings.

## Methods

### Data preparation

We searched the literature from PubMed (The National Library of Medicine), Web of Science (Clarivate), and Scopus (Elsevier) using queries for MAFLD and its possible full spellings on 10 October 2023. The exported record files were imported into R (4.4.0) and RStudio (2024.04.2) using bibliometrix package (4.2.3) [24]. We selected publications falling under the broad categories of article, review, and editorial, written in English, published from 2020 onwards, and containing exactly matched terms for MAFLD in the titles or abstracts. Publications related to NAFLD and non-alcoholic steatohepatitis (NASH) and published between 2016 and 2019 were collected from the same three databases and selected similarly.

### Publication metrics

The metrics evaluated included publication counts, publishing journals, author keywords, citation information, and author countries. All publications were classified into either article, review, or editorial using the publication types recorded in the database. Briefly, an article is an original work including studies and case reports. Review contains narrative review, systematic review and meta-analysis, and guideline. Editorial comprises comments, commentaries, letters, and other forms of short reports. The discrepancies in publication types between different databases were resolved if possible. In author keyword analysis, fully equivalent terms with different expressions were unified into a single term. Since only Web of Science and Scopus record citation information, including citation counts and reference lists, the literature from these two databases was used for citation analysis. As these databases report citation counts differently [25], the recorded citation counts were analyzed separately for Web of Science and Scopus. For each publication, we counted the citations from the MAFLD literature included in the study. Using citation information of MAFLD literature, the annual citations for representative publications and annual counts of author keywords in cited references were evaluated. Country-based publication outputs were counted by fractional counting as previously described [25]. All distinct author countries listed in each publication received equally fractionated weights summing up to 1; the sum of the counts was calculated for each country or territory [25].

### Bibliographic network

For bibliographic network analysis, the study constructed three undirected weighted networks, a co-occurrence network of author countries and keywords, as well as a co-citation network of publications [24, 26]. These components of the networks are called nodes (circles), and two related nodes are connected by edges (lines) based on several criteria. In each co-occurrence network, two countries or keywords are connected when they are indexed in the same publication. In keyword co-occurrence network analysis, author keywords in 10 or more publications are used. In co-citation network, two publications are linked when they are cited together by another publication [24, 26]. In these networks, each edge is assigned to a weight calculated as the sum of co-occurrence or co-citation times between two nodes as previously described using full counting method [24, 26]. The node strength is defined by the sum of weights of all edges attached to each node [27] and is used to assess the importance of the node. Following network construction, the communities, or groups of closely connected nodes in the networks are detected by Leiden algorithm [28].

## Results

### Broad publishing activity on MAFLD

**Fig. 1:**
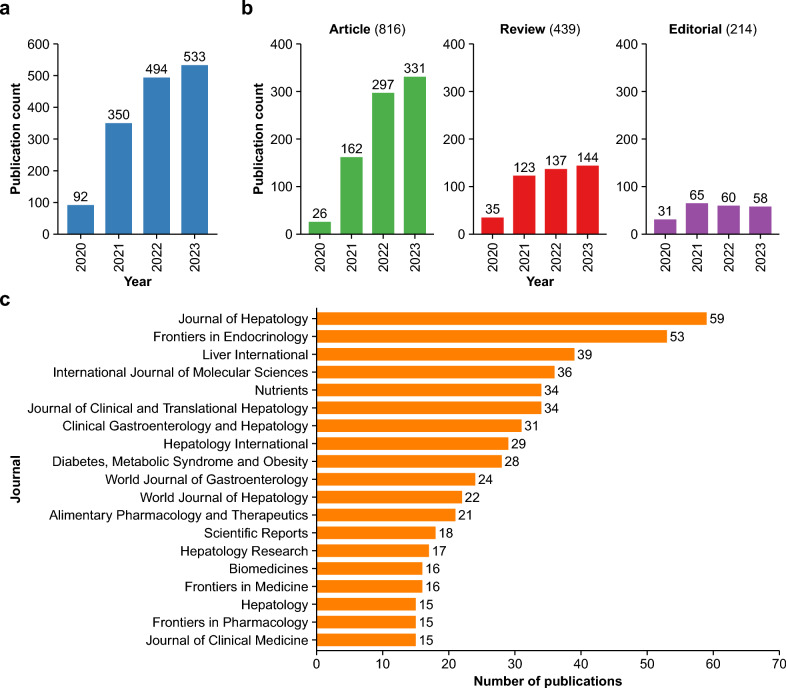
MAFLD literature and the publication journals. **a** The annual number of MAFLD papers from 2020 and 2023. **b** The annual number of MAFLD papers according to the three publication types. **c** Top journals publishing a high number of MAFLD papers

The literature search found 1469 MAFLD papers published in 434 journals (Fig. 1a, S1). The number of publications showed a steady annual increase with 533 solely in 2023 at the time of data collection (Fig. 1a). Based on the analysis of publication type, the steady increase in article number over time suggests ongoing research interest in MAFLD (Fig. 1b). The categories of journals publishing a high number of papers were not limited to gastroenterology and hepatology but included other disciplines such as endocrinology, metabolism, and general medicine and science (Fig. 1c).

### MAFLD is closely associated with metabolic dysfunction in author keywords

**Fig. 2:**
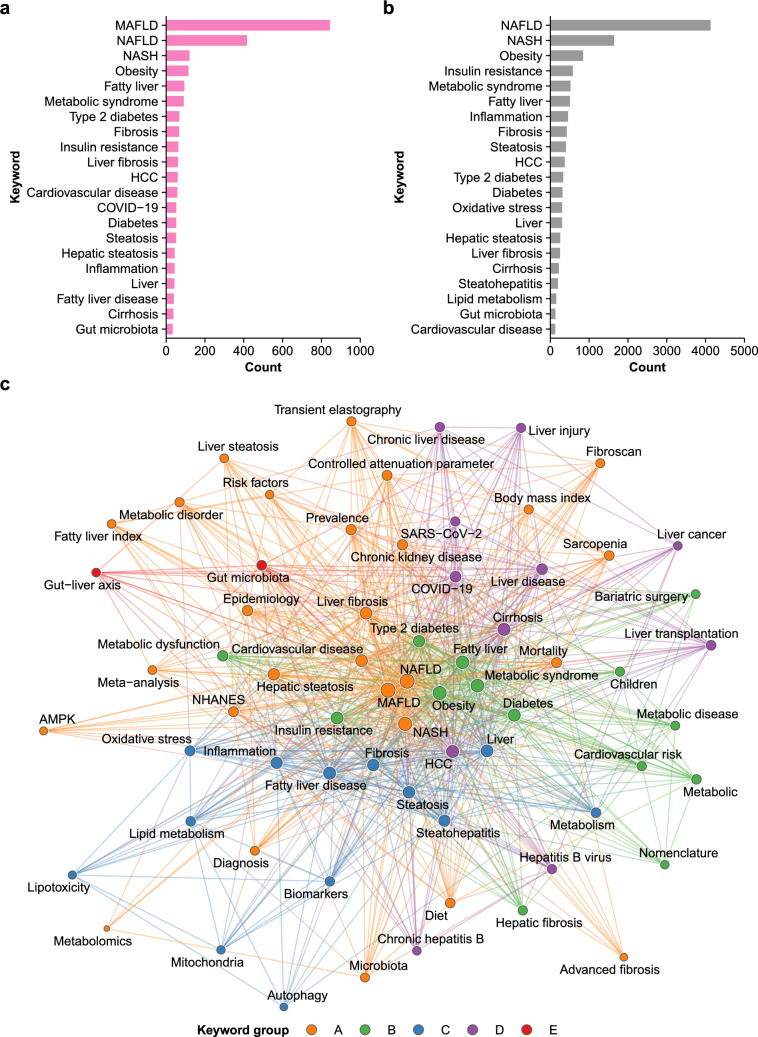
Author keywords in MAFLD and NAFLD literature. **a** Frequent author keywords in the MAFLD literature. **b** Frequent author keywords in the NAFLD literature during 2016 and 2019. **c** Co-occurrence network of 67 common author keywords in the MAFLD literature with 5 keyword groups. AMPK, adenosine monophosphate-activated protein kinase; COVID-19, coronavirus disease 2019; HCC, hepatocellular carcinoma; MAFLD, metabolic (dysfunction)-associated fatty liver disease; NAFLD, non-alcoholic fatty liver disease; NASH, non-alcoholic steatohepatitis; NHANES, National Health and Nutrition Examination Survey; SARS-CoV-2, severe acute respiratory syndrome coronavirus 2

Research topics in MAFLD were assessed using author keywords. In 1195 papers with author keywords (Fig. S1), the most common were MAFLD and other terms for fatty liver disease (Fig. 2a), which is consistent with the scope of the literature search. These were followed by terms related to metabolic dysfunction, including obesity, metabolic syndrome, (type 2) diabetes, insulin resistance, and CVD (Fig. 2a). Another category of frequent keywords included liver pathology and complications from MAFLD, such as steatosis, inflammation, fibrosis, cirrhosis, and hepatocellular carcinoma (HCC) (Fig. 2a). COVID-19 was listed as a common keyword, suggesting the influence of the pandemic on the field (Fig. 2a).

To compare the research topics between NAFLD (2016–2019) and MAFLD (2020 onwards), common keywords were analyzed for 7568 NAFLD papers published during that period (Fig. 2b, S2). Apart from the terms for fatty liver disease, the most frequent keywords related to metabolic dysfunction, as in MAFLD (Fig. 2b). This finding confirms that metabolic dysfunction was recognized as an underlying association of NAFLD prior to the proposal of MAFLD. In contrast, a major difference between NAFLD and MAFLD papers was that CVD has become one of the common keywords in the MAFLD literature (Fig. 2b). This finding implies increased awareness of cardiometabolic outcomes in MAFLD, as compared to NAFLD.

To examine for associations between keywords, a co-occurrence network of 67 common keywords was constructed (Fig. 2c). Community detection analysis of the network identified 5 keyword groups (A to E) according to the number of included terms (Fig. 2c). The largest, group A contained terms for fatty liver disease, including MAFLD. Other common terms in this group were associated with epidemiological metrics such as prevalence, mortality, and risk factor, as well as diagnostic tools for fatty liver disease, including fatty liver index and transient elastography [1]. Moreover, this group comprised CVD and CKD, two major complications of MAFLD [1, 4, 5]. Thus, group A was the central community in the network with a focus on clinical epidemiology. Group B was enriched in features of metabolic dysfunction, including obesity, (type 2) diabetes, insulin resistance, and metabolic syndrome. Group C included histological findings of MAFLD such as steatosis, steatohepatitis, inflammation, and fibrosis [29]. This group contained multiple terms for molecular mechanisms of MAFLD, including autophagy, lipotoxicity, mitochondria, and oxidative stress [29–32]. Thus, group C related to the pathophysiology of MAFLD. Group D comprised COVID-19 and severe acute respiratory syndrome coronavirus 2 (SARS-CoV-2), as well as hepatic complications such as cirrhosis, liver cancer, and HCC. The smallest group E contained gut microbiota and gut-liver axis, which play important roles in the pathogenesis of MAFLD [20, 31, 32]. Altogether, the major focus of MAFLD was clinical epidemiology and its relationship with metabolic dysfunction, whereas other domains of research included disease mechanisms, hepatic complications, and associations with COVID-19.

### Citation analysis uncovers research evolution in MAFLD

**Fig. 3:**
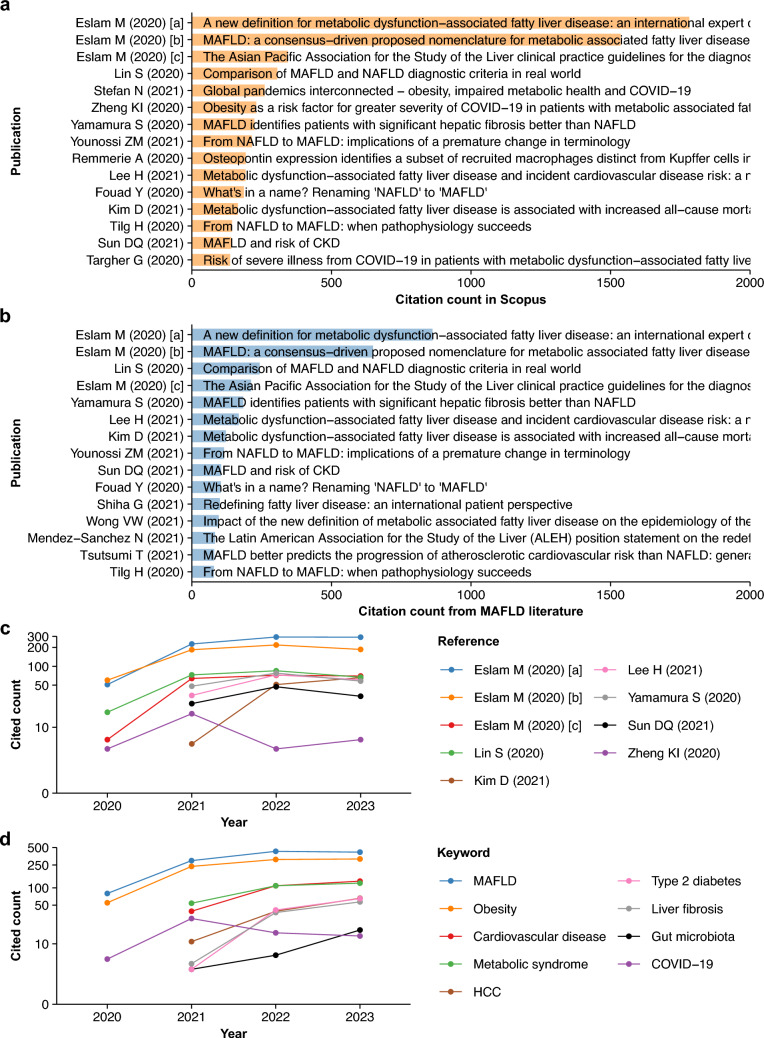
Citation analysis of the MAFLD literature. **a** Highly cited MAFLD papers recorded in the Scopus database. **b** Top publications with high citations from the MAFLD literature. **c** Annual citation counts of representative papers from MAFLD literature. **d**. Annual counts of representative keywords in cited MAFLD literature. Note that the vertical axes in **c** and** d** are log-transformed to facilitate visualization. COVID-19, coronavirus disease 2019; HCC, hepatocellular carcinoma; MAFLD, metabolic (dysfunction)-associated fatty liver disease

Citation analysis was performed to gain insights into the research findings from 1418 MAFLD papers with citation records (Fig. S1). To this end, highly cited publications were identified based on citation counts in the databases (Fig. 3a, S3) and those from the MAFLD literature included in this study (Fig. 3b) [1–3, 6–9, 11, 16, 33–42]. The two most cited publications were the initial statements proposing MAFLD in 2020 with more than 1500 overall citations [2, 3]. The highly cited publications mostly pertained to discussion of the definition [1, 6–9, 11] and validation of the criteria [33, 35, 37–39, 41, 42]. These studies show that individuals diagnosed as MAFLD or NAFLD are concordant but not fully equivalent [33, 35, 37–39, 41, 42]. Moreover, the risks of hepatic and extrahepatic complications, including liver fibrosis [35], CVD [37, 42], CKD [39], and all-cause mortality [38], were greater in MAFLD than in NAFLD. Several publications with high citation counts reported the association between MAFLD and COVID-19 (Fig. 3a) [16, 34, 40].

In representative highly cited publications related to the redefinition [1–3] and original studies investigating the clinical outcomes of MAFLD [33–35, 37–39], annual citation counts received from MAFLD papers showed overall increases during the search period (Fig. 3c). In addition, frequent keywords in cited MAFLD papers showed a similar annual increasing trend (Fig. 3d). The exceptions were papers and keywords related to COVID-19, which showed a peak in 2021 with a subsequent decrease in counts (Fig. 3c, d).

**Fig. 4:**
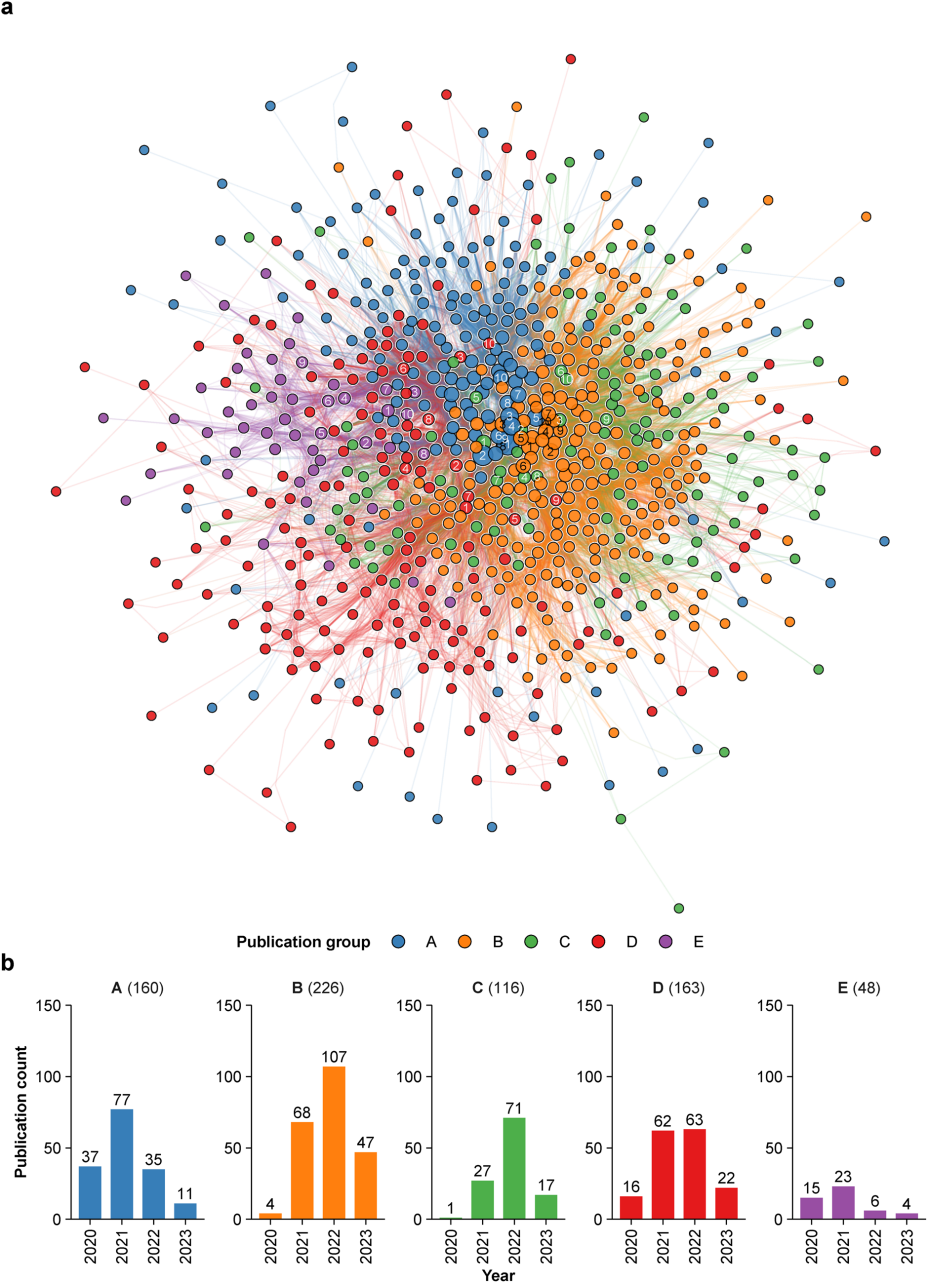
Co-citation network of MAFLD literature. **a** Co-citation network of 713 MAFLD papers in 5 major publication groups. The node size corresponds to node strength. The annotated numbers in the nodes indicate the reference number of key publications in Fig. 5. **b** The annual number of papers in each publication group of the co-citation network. The number in each group label indicates the total number of manuscripts in the publication group

**Fig. 5:**
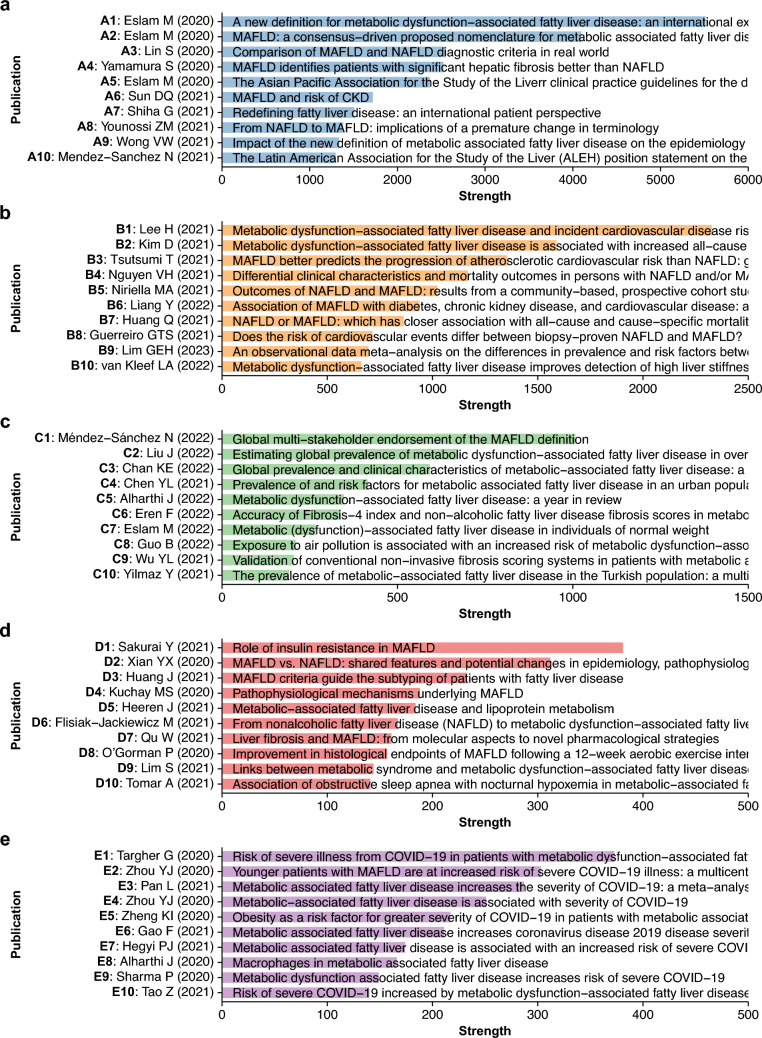
Key publications in the co-citation network of MAFLD literature. **a**–**e** 10 Key publications in each publication group determined by high node strength in the co-citation network of the MAFLD literature. Each reference number of the publication corresponds to the annotated number for the node in the co-citation network of Fig. 4a

To assess the relationships between publications, we constructed a co-citation network of 722 MAFLD papers (Fig. S1). Community detection analysis in this network divided 713 papers into 5 major publication groups (A to E) (Fig. 4a). Subsequently, 10 key publications in each group were identified based on high strength of the nodes to infer the central research findings (Fig. 5a–e).

Group A was mainly associated with the redefinition of fatty liver disease (Fig. 5a) and comprised publications with high citations (Fig. 3a, b) [1–3, 8, 9, 11, 33, 35, 39, 41]. This group included the two initial proposals for MAFLD [2, 3], which were followed by endorsements or arguments in this context [1, 8, 9, 11]. In addition, several early clinical studies demonstrated better performance of the MAFLD criteria for detecting the risk of complications, such as hepatic fibrosis [33] and CKD [39] than NAFLD.

The main topic in Group B was clinical outcomes of MAFLD (Fig. 5b) [37, 38, 42–49]. Key publications were clinical studies demonstrating that MAFLD is more strongly associated with higher risks of CVD, CKD, and all-cause mortality than NAFLD [37, 38, 42–45]. Moreover, the importance of concomitant liver diseases was suggested by findings that the clinical course of MAFLD is affected by chronic hepatitis virus infection and excessive alcohol consumption [46, 49]. As an aside, a few publications demonstrated a greater association with liver fibrosis of MAFLD than NAFLD [47, 48].

Group C covered other epidemiological features of MAFLD, including prevalence, risk factors, and non-invasive fibrosis assessment (Fig. 5c) [13, 14, 50–57]. The global prevalence of MAFLD is more than 30% of the general population [50] and is even higher in overweight and obese adults [51], indicating a substantial health burden. However, MAFLD can occur in people who are underweight or of normal weight with metabolic dysfunction and is likely to adversely affect their prognosis [50, 52, 54]. Although conventional non-invasive scoring systems for fibrosis assessment are applicable to MAFLD, they might need reevaluation in certain situations [53, 56].

Group D covered miscellaneous topics on the basic and clinical aspects of MAFLD (Fig. 5d) [29–32, 58–63]. This group included several reviews on the metabolic and molecular mechanisms of MAFLD, suggesting pathophysiology as one of the principal topics [29–32]. Group E was highly enriched in COVID-19 (Fig. 5e) [34, 40, 64–71]. Multiple clinical studies [34, 40, 64, 66, 67] and meta-analyses [65, 68, 71] in this group reported MAFLD as a risk factor for severe COVID-19.

To confirm the validity of those central topics in the co-citation network, frequent author keywords were analyzed for each publication group (Fig. S4). Whereas the terms for fatty liver disease, hepatic complications, and metabolic dysfunction were common in all groups, each group had several characteristic keywords. These included nomenclature (A), CVD and mortality (B), epidemiology (C), mitochondria and oxidative stress (D), COVID-19, SARS-CoV-2, and severity (E). These keywords are consistent with the topics of key publications in each group. Taken together, these findings support the results of the co-citation network analysis.

The annual number of MAFLD papers in each publication group was counted to assess for research trends (Fig. 4b). Group A (MAFLD definition) and E (COVID-19) had a peak in 2021 with a subsequent decline. In contrast, the number of publications in group B (clinical outcome) and C (epidemiology) showed steady increases from 2020 to 2022. Of note, all groups had less publications in 2023 likely because recent publications had limited time to receive citations by the time of analysis and less chances for co-citations to occur [25]. Together with the results of trends in citation and keyword counts (Fig. 3c, d), the findings suggest that while the redefinition of fatty liver disease was the initial focus, research has shifted to more detailed epidemiological characterization with a focus on cardiometabolic disease. On the other hand, COVID-19 in relation to MAFLD became less popular likely due to abatement of the pandemic.

In summary, citation analysis not only detected important findings but also revealed evolution of the research on MAFLD. The finding that MAFLD has high global prevalence and greater association with clinical outcomes than NAFLD validated the clinical usefulness of the definition.

### MAFLD research is driven by regional and global contributions

**Fig. 6:**
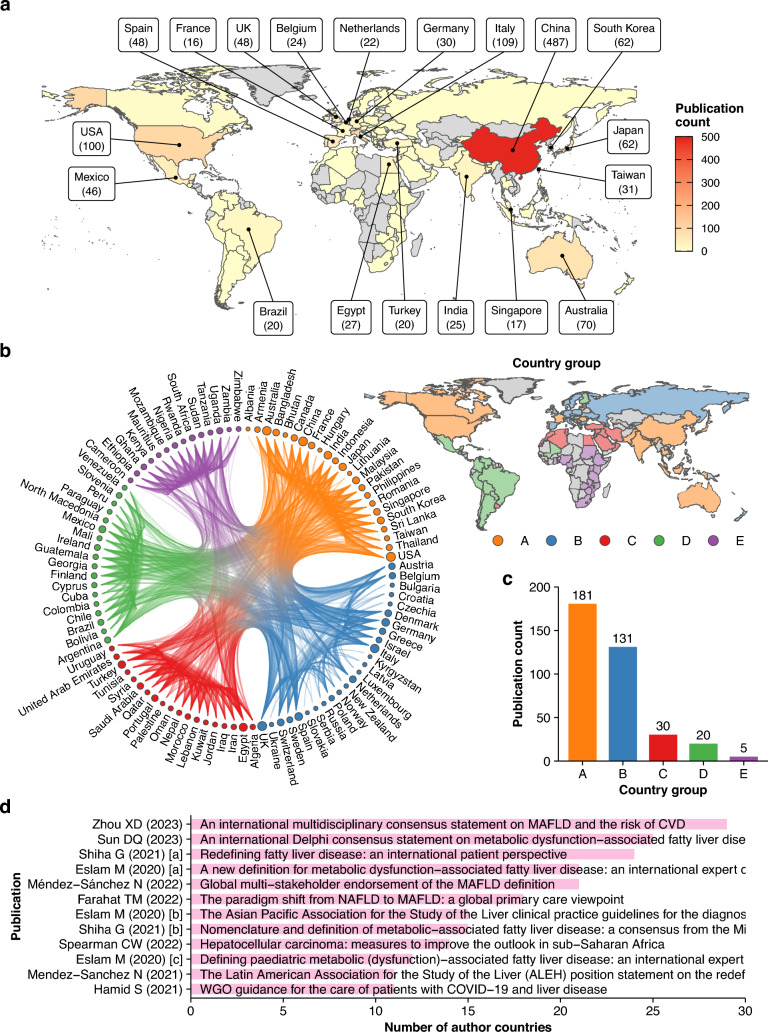
Country-based production of MAFLD literature. **a** World map showing the number of MAFLD manuscripts from each country. Countries with high publication outputs are annotated with the total number of manuscripts. **b** Co-occurrence network of 99 author countries in 367 MAFLD manuscripts from multiple countries with 5 country groups. The size of each node corresponds to the node strength. The world map shows the geographical distribution of the country groups. **c** The total number of multinational publications produced by each country group. **d** Top publications with a high number of author countries

To evaluate MAFLD research activity further, the author countries were analyzed. The 1469 papers were generated from a total of 99 countries (Fig. 6a). Of these, 1102 were from 50 single countries, whereas 367 had authors from multiple countries totaling 99 countries. The countries were located in various regions, including Asia–Pacific, Europe, North and South America, Middle East, and Africa (Fig. 6a, S5). To assess international cooperation on MAFLD research, a country co-occurrence network was constructed from the 367 multinational author papers (Fig. 6b). Community detection analysis revealed 5 country groups (A to E) according to the publication outputs (Fig. 6b, c). Each country group was predominantly distributed by world regions, such as Asia–Pacific and North America (A), Europe (B), Middle East and South Africa (C), Latin America (D), and sub-Saharan Africa (E) (Fig. 6b). However, this network also revealed numerous connections between countries from different groups (Fig. 6b), suggesting active cooperation unconstrained by geography.

### Continued international effort in advancing MAFLD for improved patient care

Considering the extent of international cooperation on MAFLD research, we sought to identify the characteristics of publications from a large number of countries. These publications and statements related to different aspects of MAFLD (Fig. 6d) [1, 2, 4, 5, 8, 10, 11, 13, 15, 72–74]. The original MAFLD definition [2] and subsequent counterparts in pediatrics [74] involved 21 and 12 countries, respectively. Consequently, various international liver societies [1, 8, 10, 11] and global stakeholders [13] expressed endorsement of MAFLD. In addition, a few publications from multiple countries discussed the benefits of MAFLD from perspectives outside the hepatologist community [8, 72]. Their central argument was that the clear name and simple criteria of MAFLD can improve overall medical care [8, 10, 72]. From the perspective of patients, the nomenclature is suggested to enhance disease understanding and alleviate stigma [8]. MAFLD can also promote multidisciplinary approaches by increasing disease awareness among healthcare providers and policy makers, including primary care physicians who play an essential role in initial screening for the disease and management of other metabolic risk factors [8, 72]. Indeed, statements on the management of CVD [4] and CKD [5] in MAFLD published in 2023 are a clear illustration of multidisciplinary cooperation among a wide group of experts from multiple countries. Notably, many publications in this listing are also identified in the preceding citation analysis (Fig. 3, 5), implying great attention to these issues [1, 2, 8, 11, 13].

## Discussion

This study based on publication metrics illustrates the overall research landscape in MAFLD from its inception. The finding of metabolic dysfunction as a crucial aspect of NAFLD corroborated the discrepancy between its definition and pathophysiology, providing further support for the redefinition. Despite being derived from consensus [2, 3], the redefinition to MAFLD was associated with significant publications and citations impacts. Consistent with its definition, the major focus in MAFLD concerns the relationships with metabolic dysfunction and an increasing focus on cardiometabolic outcomes. Despite the short period since the term was proposed, the research landscape has evolved from discussing the definition to detailed clinical characterization. MAFLD had stronger associations with both hepatic and extrahepatic complications than NAFLD, which validates the clinical usefulness of the criteria [33, 35, 37–39, 42, 44–46, 48, 49]. The clinical course of MAFLD can be modified by concomitant liver diseases such as chronic viral hepatitis and excessive alcohol consumption [33, 46, 49, 75]. These findings not only highlight the importance of considering dual etiologies, but also underscore the need for taking a holistic approach to liver health [4, 5]. These accomplishments arose from participation and collaboration across multiple countries. The value of our results is to provide a quantitative measure supporting those advancements arising from MAFLD.

The research topics in MAFLD were highly enriched for metabolic dysfunction and cardiometabolic outcomes. This focus is possibly due to adopting the term “metabolic” in the nomenclature and including cardiometabolic risk factors in the criteria. Although these changes would have increased awareness of MAFLD as a part of cardiometabolic disease, their impact could have been so large that it could have led to masking of other important topics in MAFLD. For example, within the hepatic complications of MAFLD, specific findings related to HCC were less clearly identified as compared to liver fibrosis. HCC related to MAFLD is rapidly increasing though surveillance rates are low, and these tumors are and discovered at later stages [76–78]. Although MAFLD is associated with a higher risk of HCC overall [79], the majority of HCC patients with MAFLD have concomitant liver disease such as chronic viral hepatitis or excessive alcohol consumption [78, 80, 81]. In this setting, MAFLD alone or together with other concurrent liver diseases has differential effects on HCC outcomes [78, 82–85]. In particular, MAFLD shows improved survival with lenvatinib therapy [85]. It is also of interest whether the efficacy of immunotherapy is affected by MAFLD, based on previous studies suggesting better survival with lenvatinib as compared to immunotherapy with atezolizumab and bevacizumab in NAFLD [86, 87]. Collectively, MAFLD not only plays a role as an underlying HCC etiology, but also stresses the importance of considering concomitant liver disease in the context of HCC. More detailed findings in this field are reviewed elsewhere [88].

MAFLD is accompanied by a wider variety of extrahepatic manifestations and complications than our results identified. Although underlying metabolic factors are included in the diagnostic criteria, different metabolic phenotypes in MAFLD exhibit different clinical characteristics [59, 89–91]. In particular, the presence of metabolic factors, together with concomitant liver disease, aid in stratifying the clinical outcomes of MAFLD, including fibrosis, cardiovascular events, and mortality [46, 59, 89]. Conversely, MAFLD predicts the future development of incident metabolic dysfunction and CVD [44, 92]. Within the spectrum of cardiometabolic complications, the associated diseases that have been characterized in more detail include coronary artery disease [93] and end-stage kidney disease [94]. In addition, MAFLD has association with various extrahepatic malignancies including gastrointestinal cancers [95–98]. Other associated conditions of MAFLD include obstructive sleep apnea [63], hypothyroidism [99], reflux esophagitis [100], and chronic obstructive pulmonary disease [101]. More comprehensive descriptions of the extrahepatic complications of MAFLD are reviewed elsewhere [102, 103]. While many of these associations have been demonstrated for NAFLD [104], the findings suggest the critical value of the redefinition to MAFLD.

Due to the search period as well as limitations of citation analysis in detecting the recent findings [25], our results did not reveal findings about MASLD in relation to MAFLD. Our results on MAFLD demonstrated their clinical utility in improved detection of both hepatic and extrahepatic outcomes. From this perspective, similar to MAFLD, MASLD should undergo a similar validation process before full acceptance to ensure clinical usefulness. At the time of writing, studies investigating the clinical characterization and the performance of MASLD have begun to accumulate [105–114]. However, as three different criteria now exist, each study has different study designs, whether analyzing MASLD alone [109, 110] or comparing it with the other one or two criteria [105–108, 111–114]. The available results suggest limitations of MASLD in predicting clinical outcomes [105–108, 110, 114]. In the current situation, it would, therefore, be premature to evaluate the clinical utility of the MASLD criteria as compared to MAFLD [115, 116].

The progress on MAFLD was driven by global commitments from a wide variety of stakeholders. Thus, it is critical to consider different perspective in the setting of a disease with a high global prevalence [50, 51]. After the proposal of MASLD, it was pointed out that unfamiliarity with the term “steatotic” can hinder understanding and communication with patients and healthcare professionals other than hepatologists [117, 118]. As shown in our results [8, 10, 13, 72] together with other reports [119, 120], this situation is in contrast to the debate between NAFLD and MAFLD, in which a clear disease name with simple criteria was emphasized. In multiple published discussions, whereas “non-alcoholic” in NAFLD is considered inappropriate both in terms of pathophysiology and stigma [8, 72, 120], the problem with “fatty liver” has not been pointed out [120]. This finding is consistent with a recent global survey reporting that most patients perceive fatty liver disease as a neutral term [121]. In addition, many languages do not have terms that differentiate “fatty” and “steatotic” [122–124]. Indeed, due to the linguistic issues and clinical usefulness, multiple national and regional communities continue to endorse fatty liver disease [124] or MAFLD [123] even after the MASLD proposal. These findings highlight the importance of careful consideration before changing the nomenclature of fatty liver disease from a global perspective.

As compared to the previous similar studies in the field [19–23], our methods involving the collection of records from multiple databases and heavy reliance on programming language allowed comprehensive, flexible, and detailed analysis of the literature pertaining to MAFLD. However, this study also has several limitations that have not mentioned previously. First, as the primary source of data, the databases we used are known to contain indexing errors including incorrect reference information [25]. Therefore, undetected errors may have led to inaccurate results. Moreover, the quality of publications was not assessed for inclusion in this study. Another limitation relates to our literature selection strategy which required the terms for MAFLD in the title or abstract. Although the intention was to retrieve publications specifically addressing the topic, this procedure could have excluded literature lacking the search terms. This is particularly the case with editorials with concise titles and no abstract [12]. Conversely, MAFLD was not the direct interest in some papers where the term is used in relation to another topic such as HCC [73] or COVID-19 [15, 16]. A final caveat is that citation does not necessarily reflect the scientific importance or validity of the publication [125]. Within these limitations, our results convey an objective overview of MAFLD research to date.

In conclusion, this survey provides a quantitative measure that highlights the considerable global research contribution to discussing and validating the MAFLD definition and establishing its role as a part of cardiometabolic disease. Although this is a crucial first step, the field needs to accelerate basic and clinical research that can help improve disease understanding and patient management.

## Supplementary Information

Below is the link to the electronic supplementary material.

Supplementary file1 (PDF 469 kb)

## Data Availability

The detailed methods for the literature search and data analysis are described in Supplementary material. The R code for data analysis is deposited in GitHub (https://github.com/maitosuoh/mafld-bibliometrics).

## Abbreviations

AMPK: Adenosine monophosphate-activated protein kinase
CKD: Chronic kidney disease
COVID-19: Coronavirus disease 2019
CVD: Cardiovascular disease
HCC: Hepatocellular carcinoma
MAFLD: Metabolic (dysfunction)-associated fatty liver disease
MASLD: Metabolic dysfunction-associated steatotic liver disease
NAFLD: Non-alcoholic fatty liver disease
NASH: Non-alcoholic steatohepatitis
NHANES: National Health and Nutrition Examination Survey
SARS-CoV-2: Severe acute respiratory syndrome coronavirus 2
