# Supplementary material for: Metabolic (dysfunction)-associated fatty liver disease metrics and contributions to liver research
Source: Hepatol Int. 2024 Oct 16;18(6):1740–55. doi: 10.1007/s12072-024-10731-0 (PMC11632019; doi:10.1007/s12072-024-10731-0)
Supplement: Supplementary file 1 — Supplementary file1 (PDF 469 kb) [file 12072_2024_10731_MOESM1_ESM.pdf]

Supplementary material

Title: Metabolic (dysfunction)-associated fatty liver disease metrics and contributions to liver research

Journal: Hepatology International

Authors: Maito Suoh[1], Saeed Esmaili[1], Mohammed Eslam[1], Jacob George[1]

Affiliation: 1 Storr Liver Centre, The Westmead Institute for Medical Research, Westmead Hospital and The University of Sydney, Westmead, New South Wales, Australia

Corresponding author: Jacob George
E-mail: jacob.george@sydney.edu.au

Supplementary methods

Database search

The literature related to MAFLD was searched from PubMed, Web of Science, and Scopus. Pubmed (The National Library of Medicine), Web of Science (Clarivate), and Scopus (Elsevier) using the following queries on 10 October 2023.

The literature related to non-alcoholic fatty liver disease (NAFLD) and non-alcoholic steatohepatitis (NASH) were collected from the same three databases with the queries for possible full spellings of NAFLD and NASH together with the abbreviation "NAFLD" during the revision on 21 July 2024. The abbreviation "NASH" was excluded from the query to avoid non-specific matching. The inclusion period for NAFLD papers is set between 2016 and 2019 to show the previous research status in this field. For this purpose, the records between 2015 and 2020 were exported from each database in a single file or in batches to add one extra one-year margin before and after the defined search period.

Pubmed

((((((((("MAFLD") OR ("METABOLIC-DYSFUNCTION-ASSOCIATED FATTY LIVER DISEASE")) OR ("METABOLIC-(DYSFUNCTION)-ASSOCIATED FATTY LIVER DISEASE")) OR ("METABOLIC-DYSFUNCTION ASSOCIATED FATTY LIVER DISEASE")) OR ("METABOLIC-(DYSFUNCTION) ASSOCIATED FATTY LIVER DISEASE")) OR ("METABOLIC DYSFUNCTION-ASSOCIATED FATTY LIVER DISEASE")) OR ("METABOLIC (DYSFUNCTION)-ASSOCIATED FATTY LIVER DISEASE")) OR ("METABOLIC DYSFUNCTION ASSOCIATED FATTY LIVER DISEASE")) OR ("METABOLIC (DYSFUNCTION) ASSOCIATED FATTY LIVER DISEASE")) OR ("METABOLIC-ASSOCIATED FATTY LIVER DISEASE")) OR ("METABOLIC ASSOCIATED FATTY LIVER DISEASE")

(((((("NAFLD") OR ("NON-ALCOHOLIC FATTY LIVER DISEASE")) OR ("NON ALCOHOLIC FATTY LIVER DISEASE")) OR ("NONALCOHOLIC FATTY LIVER DISEASE")) OR ("NON-ALCOHOLIC STEATOHEPATITIS")) OR ("NON ALCOHOLIC STEATOHEPATITIS")) OR ("NONALCOHOLIC STEATOHEPATITIS")

Web of Science

((((((((((ALL=("MAFLD")) OR ALL=("METABOLIC-DYSFUNCTION-ASSOCIATED FATTY LIVER DISEASE")) OR ALL=("METABOLIC-(DYSFUNCTION)-ASSOCIATED FATTY LIVER DISEASE")) OR ALL=("METABOLIC-DYSFUNCTION ASSOCIATED FATTY LIVER DISEASE")) OR ALL=("METABOLIC-(DYSFUNCTION) ASSOCIATED FATTY LIVER DISEASE")) OR ALL=("METABOLIC DYSFUNCTION-ASSOCIATED FATTY LIVER DISEASE")) OR ALL=("METABOLIC (DYSFUNCTION)-ASSOCIATED FATTY LIVER DISEASE")) OR ALL=("METABOLIC DYSFUNCTION ASSOCIATED FATTY LIVER DISEASE")) OR ALL=("METABOLIC (DYSFUNCTION) ASSOCIATED FATTY LIVER DISEASE")) OR ALL=("METABOLIC-ASSOCIATED FATTY LIVER DISEASE")) OR ALL=("METABOLIC ASSOCIATED FATTY LIVER DISEASE")

"NAFLD" (All Fields) or "NON-ALCOHOLIC FATTY LIVER DISEASE" (All Fields) or "NON ALCOHOLIC FATTY LIVER DISEASE" (All Fields) or "NONALCOHOLIC FATTY LIVER DISEASE" (All Fields) or "NON-ALCOHOLIC STEATOHEPATITIS" (All Fields) or "NON ALCOHOLIC STEATOHEPATITIS" (All Fields) or "NONALCOHOLIC STEATOHEPATITIS" (All Fields) and 2020 or 2019 or 2018 or 2017 or 2016 or 2015 (Publication Years)

Scopus

(TITLE-ABS-KEY ({MAFLD}) OR TITLE-ABS-KEY ({METABOLIC-DYSFUNCTION-ASSOCIATED FATTY LIVER DISEASE}) OR TITLE-ABS-KEY ({METABOLIC-(DYSFUNCTION)-ASSOCIATED FATTY LIVER DISEASE}) OR TITLE-ABS-KEY ({METABOLIC-DYSFUNCTION ASSOCIATED FATTY LIVER DISEASE}) OR TITLE-ABS-KEY ({METABOLIC-(DYSFUNCTION) ASSOCIATED FATTY LIVER DISEASE}) OR TITLE-ABS-KEY ({METABOLIC DYSFUNCTION-ASSOCIATED FATTY LIVER DISEASE}) OR TITLE-ABS-KEY ({METABOLIC (DYSFUNCTION)-ASSOCIATED FATTY LIVER DISEASE}) OR TITLE-ABS-KEY ({METABOLIC DYSFUNCTION ASSOCIATED FATTY LIVER DISEASE}) OR TITLE-ABS-KEY ({METABOLIC (DYSFUNCTION) ASSOCIATED FATTY LIVER DISEASE}) OR TITLE-ABS-KEY ({METABOLIC-ASSOCIATED FATTY LIVER DISEASE}) OR TITLE-ABS-KEY ({METABOLIC ASSOCIATED FATTY LIVER DISEASE}))

(TITLE-ABS-KEY ({NAFLD}) OR TITLE-ABS-KEY ({NON-ALCOHOLIC FATTY LIVER DISEASE}) OR TITLE-ABS-KEY ({NON ALCOHOLIC FATTY LIVER

DISEASE}) OR TITLE-ABS-KEY ({NONALCOHOLIC FATTY LIVER DISEASE}) OR TITLE-ABS-KEY ({NON-ALCOHOLIC STEATOHEPATITIS}) OR TITLE-ABS-KEY ({NON ALCOHOLIC STEATOHEPATITIS}) OR TITLE-ABS-KEY ({NONALCOHOLIC STEATOHEPATITIS})) AND PUBYEAR > 2014 AND PUBYEAR < 2021

Data export

The search records from each literature database were exported in a format compatible with the subsequent data import into R environment using bibliometrix (4.2.3) [1]. Records from PubMed were exported by “Save citation to file” option in “PubMed” format, which generates a text file. In Web of Science, all record contents were selected and downloaded by “Export” in “Plain text file”. In Scopus, all information was included and saved by “Export” with File Types “CSV”.

Data import

The downloaded file from each literature database was imported into R (4.4.0) and RStudio (2024.04.2) with bibliometrix (4.2.3). This procedure generates a data frame of literature records amenable to subsequent data handling, including manipulation, analysis, and visualisation with the relevant R packages.

Data preprocessing

Data preprocessing was performed separately for the records from each literature database. This process involved selecting eligible papers and the necessary information for analysis and assigning a unique identifier to each publication. We selected publications falling under the broad category of article, review, and editorial, written in English, published from 2020 onwards, and containing exactly matched terms for MAFLD or its all possible full spellings in the titles or abstracts. The ineligible publication types include correction and erratum, conference papers and meeting abstracts, book chapters, or any other material that does not fit in the three categories as previously mentioned in broad sense. The Digital Object Identifier (DOI), PubMed ID (PMID), and title were used to identify each publication. Possible errors in these sections were detected by checking duplicates, missing data, and inconsistent entries across different databases. These errors were mainly fixed by filling entries from other

database or resolving discrepancies between the databases. We only referred to the original documents and manually corrected the errors unless otherwise possible. As a unique identifier, we primarily used DOI followed by PMID. If both of them are unavailable, a character vector combining the first author, year, and journal name was used. After three datasets underwent individual data cleaning, they were merged into a single dataset using the assigned unique identifier. After the dataset merge, duplicates that could not be detected in the previous steps were removed. Data preprocessing was performed by the packages in tidyverse [2]. The process for literature selection is visualised in a flow chart (Fig. S1).

The literature selection in NAFLD papers were performed similarly to MAFLD papers as described above. However, the process was modified to accommodate the large number of records and a wider variety of publication types that were not present in MAFLD papers. The process for literature selection is visualised in a flow chart (Fig. S2).

Data analysis

The merged dataset contains all information from the individual dataset after preprocessing. Each database records the same information in different styles. To ensure consistency of data, the most suitable information for each analysis was selected from a primary dataset. In case of missing entries in the primary database, data from other databases were used accordingly. In most analyses, PubMed was used as a primarily source of information due to high coverage of the literature and low incompleteness of the data. However, data from Web of Science were chiefly used in country analysis because the entries were more amenable to extracting information on author country. Since citation information, including citation times and reference lists, was only recorded in Web of Science and Scopus, only records from these two databases were used for citation analysis.

Publication metrics

The publication metrics evaluated in this study include publication counts, published year, publication types, publishing journals, author countries, author keywords, and citation counts. The analysis and visualisation of these metrics was conducted by tidyverse [2] with additional packages for text annotation. Packages used for specific analysis are described below.

Published year

Data entries in PubMed were primarily used for published year. If the published year is 2024 in early access publications, it was changed from 2024 to 2023. The annual number of publications was counted and shown in a bar chart (Fig. 1a).

Publication type

In this study, all publications were classified into either article, review, or editorial based on the records in the databases. Any different entries in eligible publications were changed to the closest possible category. Article is an original work including studies and case reports. Review contains narrative review, systematic review and meta-analysis, and guideline. Editorial comprises comment, commentary, letter, and other forms of short reports.

The publication types in each database were changed to the three categories as described above. The final publication type for each literature was determined based on the agreement between different datasets. When inconsistency existed between databases, more common publication types were selected if available. After data cleaning, the annual number of publications within each publication type was counted and shown in bar charts (Fig. 1b).

Publishing journal

The journal names were mainly collected from PubMed. The number of papers in highly publishing journals was shown in a bar chart (Fig. 1c).

Author country

The information on author country was mainly retrieved from Web of Science. The missing author countries were manually filled. For each publication, all author countries are stored in a single cell delimited by ";". The cells for author countries were separated into long format to obtain all countries for each publication. After manual corrections, each indexed country name was converted into a formatted name and ISO-3 code using countrycode (1.6.0) [3].

For each publication, only distinct author countries were retained, and the number of publications was counted for each country using fractional counting as previously described [4]. If a publication is produced by authors from a single country, this country received a count of 1. In a publication from multiple countries, each distinct country was assigned to an equally fractionated counts that sum up to 1. The total publication output for each country is calculated as the sum of these two counts from all literature included in the study. The publication output from each country is shown in a world heatmap with annotations of highly productive countries

(Fig. 6a). The geospatial visualisation was performed with sf (1.0-16) [5] and geographical data from rnaturalearth (1.0.1) and CoordinateCleaner (3.0.1) [6]. The number of papers from highly productive countries is shown in a stacked bar chart with breakdown into counts of publications from single or multiple countries (Fig. S4). To illustrate international collaboration, publications from a large number of countries are shown in a bar chart with the title annotated on a bar (Fig. 6d).

Author keyword

Entries in PubMed were primarily used for author keywords. For each publication, all author keywords are stored in a single cell delimited by ";". After removal of publications without keywords, cells for keywords are separated into long format. Totally equivalent terms with different expressions were unified into a single term. For example, the abbreviations all possible variations of the full spellings for MAFLD or NAFLD were changed into a single term "MAFLD" or "NAFLD", respectively. Similarly, "diabetes" and "diabetes mellitus" were converted to "diabetes". Other formatting includes changing pleural to singular form, or vice versa. In contrast, "fibrosis" and "liver fibrosis", "fatty liver" and "hepatic steatosis", or "diabetes" and "type 2 diabetes" were treated as different terms as they were composed of different words. The complete formatting process for author keywords is described in the deposited R code. After selecting the distinct author keywords for each publication, the total number of appearances was counted and shown in a bar chart for MAFLD (Fig. 2a) and NAFLD (Fig. 2b).

Citation count

Web of Science and Scopus records citation information differently, and the total citation counts were analysed separately. The citation counts from Scopus were used to determine highly cited publications (Fig. 3a) as they were likely to be greater than those in Web of Science [4]. Highly cited papers in Web of Science are reported in Supplementary material (Fig. S2).

We also counted citation times from the MALFD literature in our dataset. To this end, the publications in reference information were extracted and matched with the literature of interest. The references in each publication are recorded in a single cell delimited by ";". After removal of publications without references, cells for references are separated into long format. As the main reference information, Web of Science records DOI, whereas Scopus indexes titles. To extract the publications of interest from the references, a set of DOI or titles was created

from the literature for citation analysis, and only references that matched them were retained with the corresponding unique identifiers assigned. Detected errors in the matched references were manually fixed. The extracted references from Web of Science and Scopus were merged into the same dataset, and only distinct reference records for each publication were counted as citation times from MAFLD literature (Fig. 3b).

The citation counts for each publication are shown in a bar chart with its title annotated on each bar (Fig. 3a, b). Using the citation counts from MAFLD literature, annual cited times of representative papers or counts of associated keywords were shown in a line chart (Fig. 3c, d).

Bibliographic network

For bibliographic network analysis, we constructed three undirected weighted networks, co-occurrence network of author country and keyword, as well as co-citation network. The data transformation for network analysis was performed with the packages in tidyverse [2]. The network analysis was performed with igraph (2.0.3) [7] and tidygraph (1.3.1), and network visualisation was implemented using ggraph (2.2.1).

Co-occurrence network analysis

Co-occurrence networks were constructed for author country and keywords using the full counting method as previously described [8]. Briefly, we first converted the data frame for distinct author countries or keywords in long format into wide format to create a matrix $\boldsymbol{A}$ with each country or keyword in rows and each publication in columns. Each element of matrix $\boldsymbol{A}$ is 1 if a country or keyword in each row is present in publication in each column and 0 otherwise. Co-occurrence matrix $\boldsymbol{B}$ was obtained by the following matrix operation.

$$\boldsymbol{B} = \boldsymbol{A}\boldsymbol{A}^{\boldsymbol{T}}$$

Each element in co-occurrence matrix $\boldsymbol{B}$ indicates the total co-occurrence times between two countries or keywords and used as edge weights in the co-occurrence network. After the construction of co-occurrence networks, the communities in the networks were detected by Leiden algorithm [9] with edge weights as weights to optimise modularity. In this study, the detected communities in country or keyword co-occurrence networks are termed country or keyword groups, respectively. The sum of weights of all edges attached to a node, or strength, is calculated to evaluate the importance of each country or keyword in the network.

Co-occurrence network of author countries was visualised by hierarchical edge bundling [10]. This method creates a network in which nodes are placed in a circular layout and connected by bundled edges inside the circle (Fig. 6b). Co-occurrence network of author keywords was displayed in force-directed algorithm of Fruchterman and Reingold (Fig. 2b) [11]. Some keywords were changed to the abbreviated forms to avoid overplotting. In both networks, the nodes were coloured by the identified communities, and each node size is scaled to the node strength.

The geographical distribution of the country groups was visualised in a world map using sf (1.0-16) (Fig. 6b) [5]. The total number of publications produced by each country groups is calculated with fractionated counting as previously described (Fig. 6c) [8].

Co-citation network analysis

Co-citation occurs when two publications are citated together by another publication. Co-citation network was constructed by full counting method as previously described [8]. To this end, we used the data frame prepared for the analysis of citation counts from the MAFLD literature. This data frame contains the relationship between citing and cited publications within our dataset. Because co-citation assumes the citing publication to have more than one references [8], publications citing only one reference were removed. The data frame showing references for each publication in long format was converted into wide format to create a matrix $\boldsymbol{C}$ with each publication of interest in rows and citing publication in columns. Each element of matrix $\boldsymbol{C}$ is 1 if a publication in the row is cited by a publication in the column and 0 otherwise. Co-occurrence matrix $\boldsymbol{W}$ was obtained by the following matrix operation.

$$\boldsymbol{W} = \boldsymbol{C}\boldsymbol{C}^{T}$$

Each element in co-citation matrix $\boldsymbol{W}$ indicates the total co-citation times between two publications of interest and used as edge weights in the co-citation network. After the construction of co-citation networks, the communities were detected by Leiden algorithm [9] with edge weights as weights to optimise modularity. In this study, the detected communities in the co-citation network are called publication groups. The strength of each node is used to evaluate the importance of the publication in the network.

The co-citation network is displayed in large graph layout (LGL) [12] with the edges bundled by a technique called edge bundling to facilitate visualisation (Fig. 4a). The nodes are coloured by the publication groups, and each node size is scaled to the node strength. The annual number of papers in each publication group is counted and shown in bar charts to visualise the chronological trends (Fig. 4b). In each publication group, top 10 publications with

high node strength are selected as key publications to infer the central research findings. These results are shown in bar charts of node strength for each key publication with the title annotated on the corresponding bar (Fig. 5a-e). To confirm the topics identified by the key publications, frequent author keywords were counted for each publication group and plotted in a bar chart (Fig. S3a-e).

Software and package

**Software**	**Version**	**Source**
R	4.4.0	https://www.R-project.org/
RStudio	2024.04.2	http://www.posit.co/
**Packages**	**Version**	**Source**
Data import		
bibliometrix	4.2.3	10.1016/j.joi.2017.08.007
Data analysis		
dplyr	1.1.4	https://CRAN.R-project.org/package=dplyr
forcats	1.0.0	https://CRAN.R-project.org/package=forcats
glue	1.7.0	https://CRAN.R-project.org/package=glue
purrr	1.0.2	https://CRAN.R-project.org/package=purrr
stringr	1.5.1	https://CRAN.R-project.org/package=stringr
tidyr	1.3.1	https://CRAN.R-project.org/package=tidyr
tidyverse	2.0.0	https://doi.org/10.21105/joss.01686
tibble	3.2.1	https://CRAN.R-project.org/package=tibble
Data visualisation		
ggplot2	3.5.1	https://ggplot2.tidyverse.org
ggrepel	0.9.5	https://CRAN.R-project.org/package=ggrepel
ggtext	0.1.2	https://CRAN.R-project.org/package=ggtext
cowplot	1.3.1	https://CRAN.R-project.org/package=cowplot
patchwork	1.2.0	https://CRAN.R-project.org/package=patchwork
Data export		
openxlsx	4.2.5.2	https://CRAN.R-project.org/package=openxlsx
Geospatial analysis		
CoordinateCleaner	3.0.1	https://github.com/ropensci/CoordinateCleaner
countrycode	1.6.0	https://doi.org/10.21105/joss.00848
rnaturalearth	1.0.1	https://CRAN.R-project.org/package=rnaturalearth
rnaturalearthdata	1.0.0	https://CRAN.R-project.org/package=rnaturalearthdata
rmapshaper	0.5.0	https://CRAN.R-project.org/package=rmapshaper
sf	1.0-16	https://doi.org/10.32614/RJ-2018-009
Network analysis		
igraph	2.0.3	https://CRAN.R-project.org/package=igraph
tidygraph	1.3.1	https://CRAN.R-project.org/package=tidygraph
Network visualisation		
ggraph	2.2.1	https://CRAN.R-project.org/package=ggraph

Data availability

The R code for the analysis was deposited in GitHub (https://github.com/maitosuoh/mafld-bibliometrics). The exported files from the literature databases are not provided due to potential copyright infringement.

Supplementary results

Supplementary figures

Fig. S1 Flow chart for selection process of MAFLD literature

Fig. S2 Flow chart for selection process of NAFLD literature

Fig. S3 a, b Highly cited MAFLD papers in Scopus (**a**) and Web of Science (**b**)

Fig. S4 a-e Frequent keywords in each publication group (A-E) of the co-citation network created from MAFLD literature

Fig. S5 Top countries producing MAFLD literature

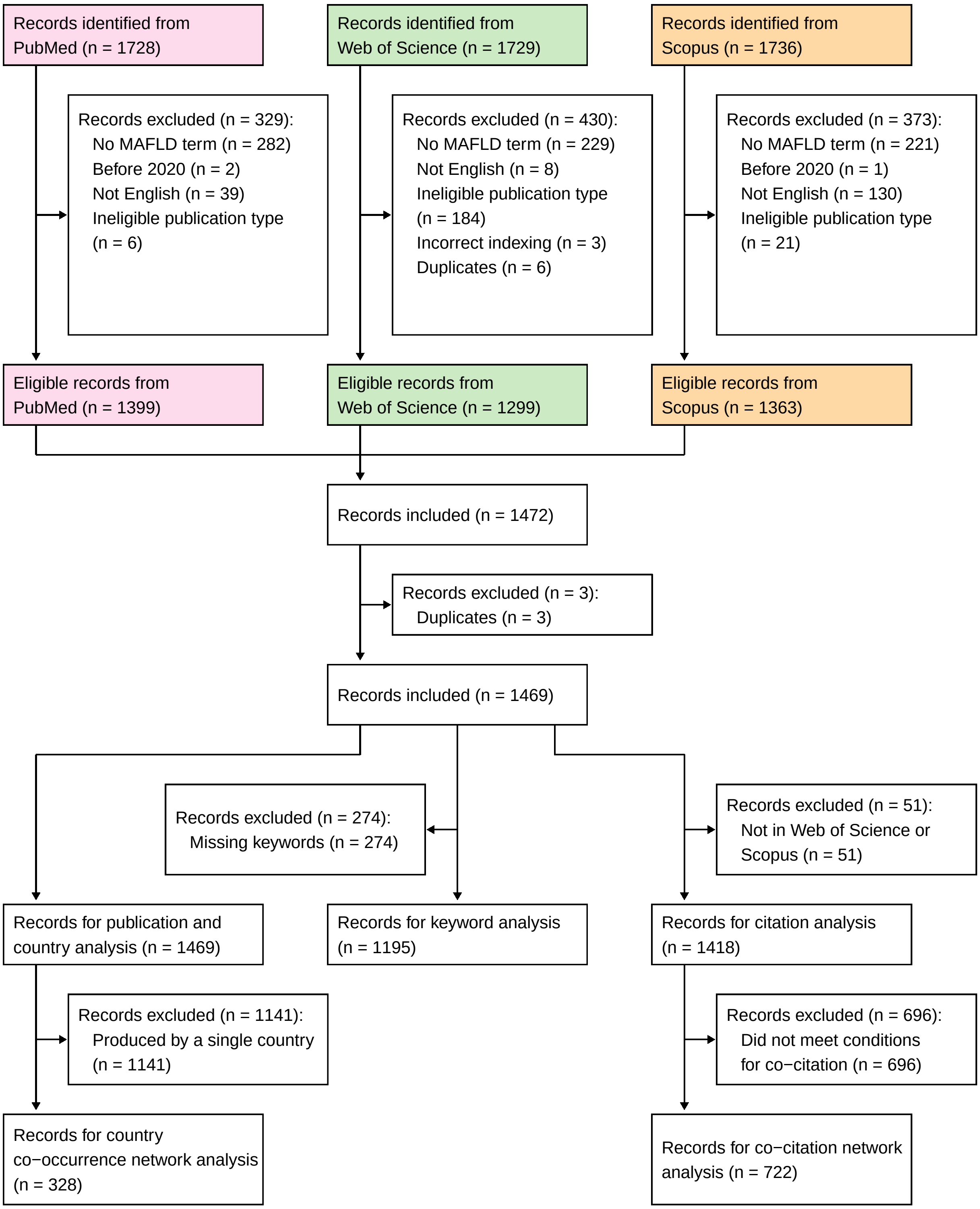

Fig. S1 Flow chart of selection process for MAFLD literature

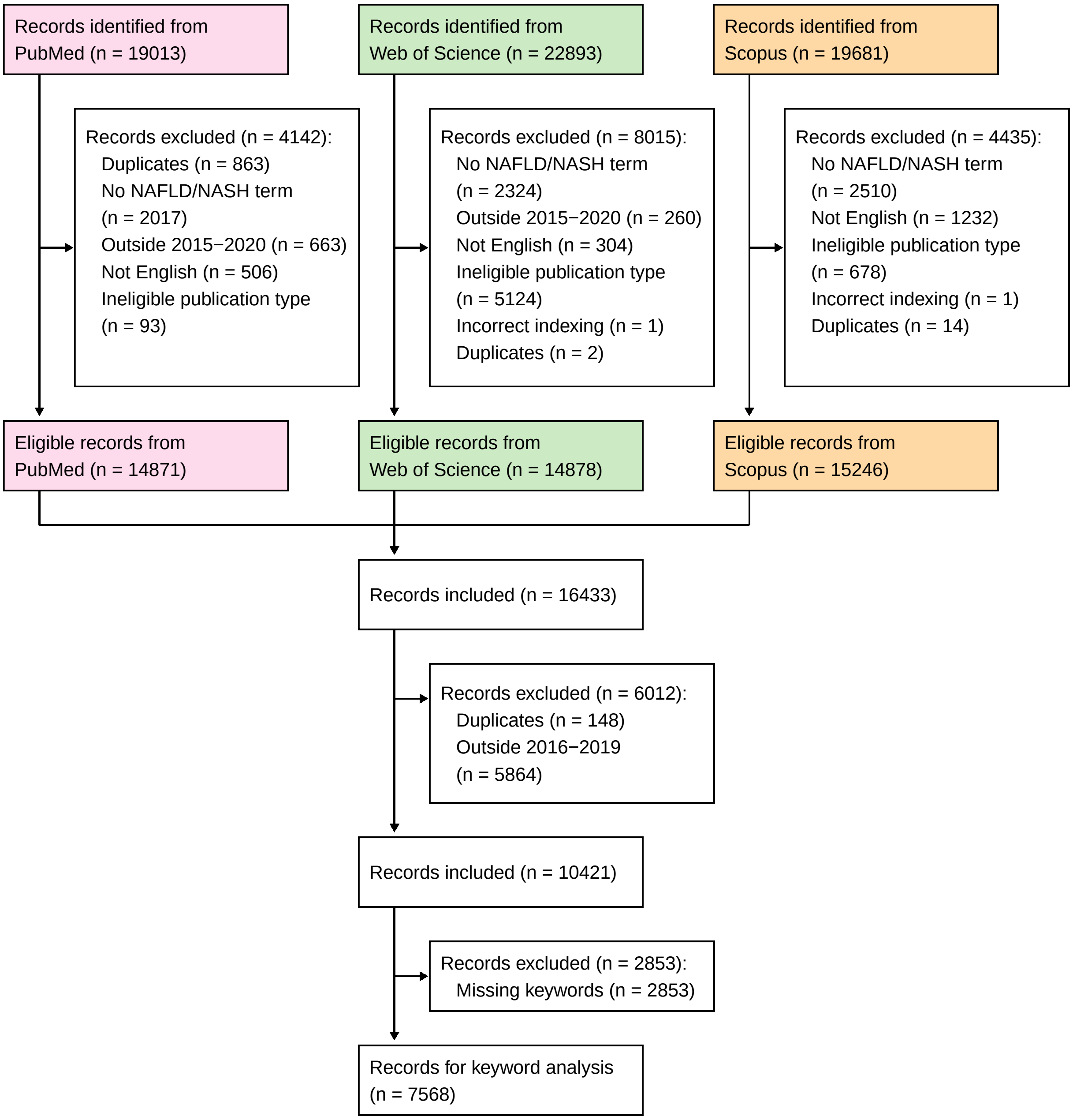

Fig. S2 Flow chart of selection process for NAFLD literature

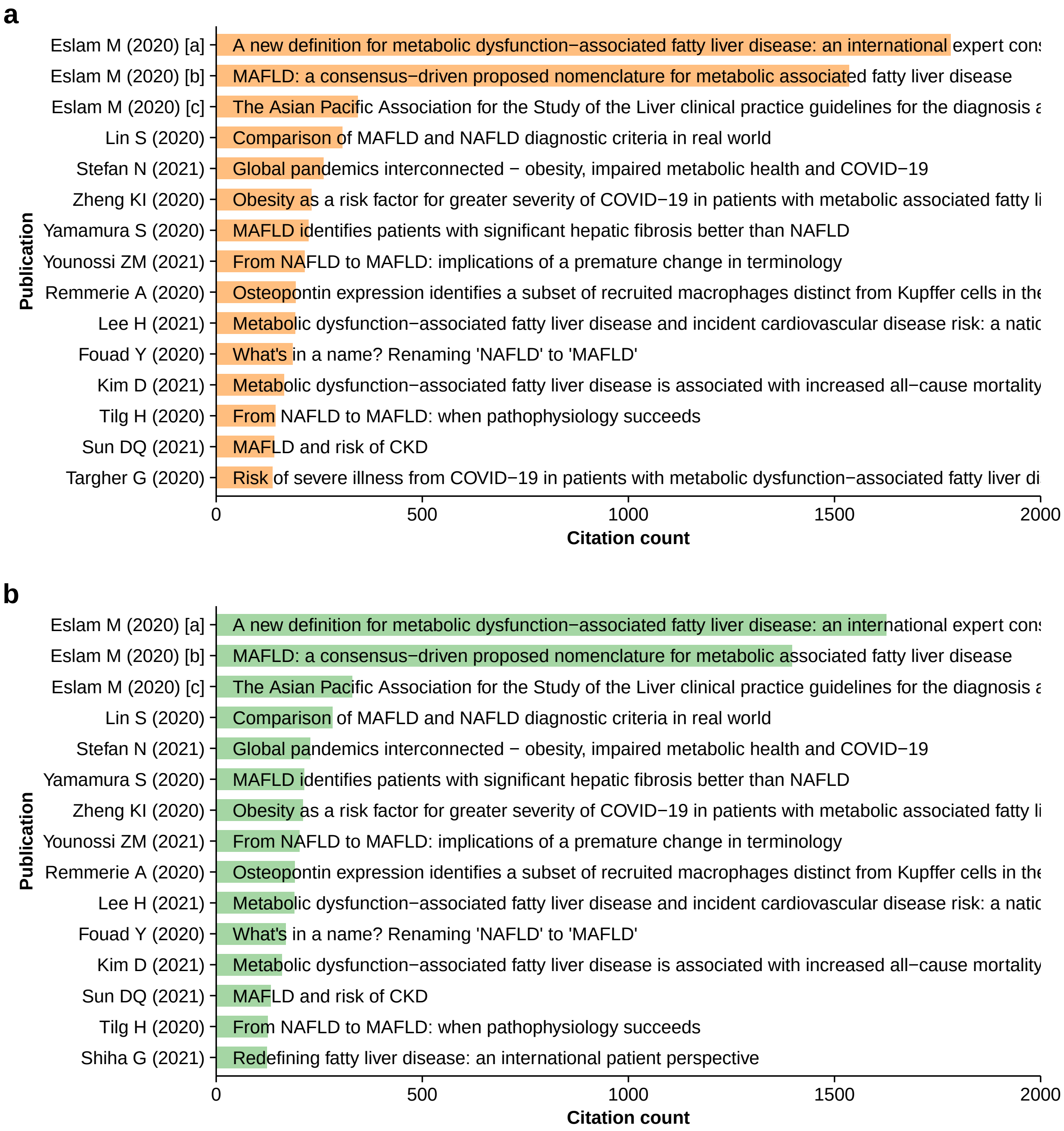

Fig. S3 a, b Highly cited MAFLD papers in Scopus (**a**) and Web of Science (**b**)

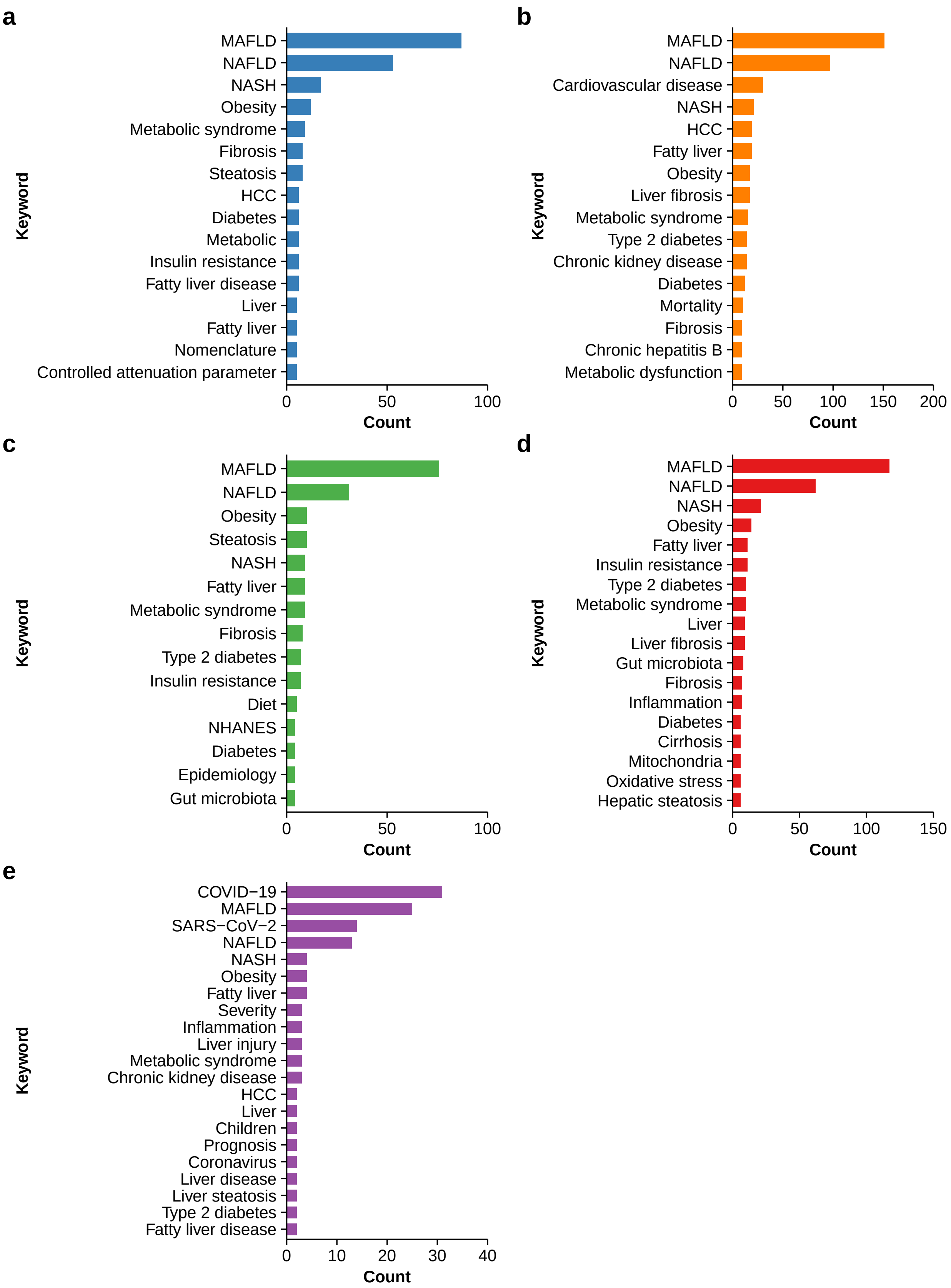

Fig. S4 a−e Frequent keywords in each publication group (A−E) of the co−citation network created from MAFLD literature

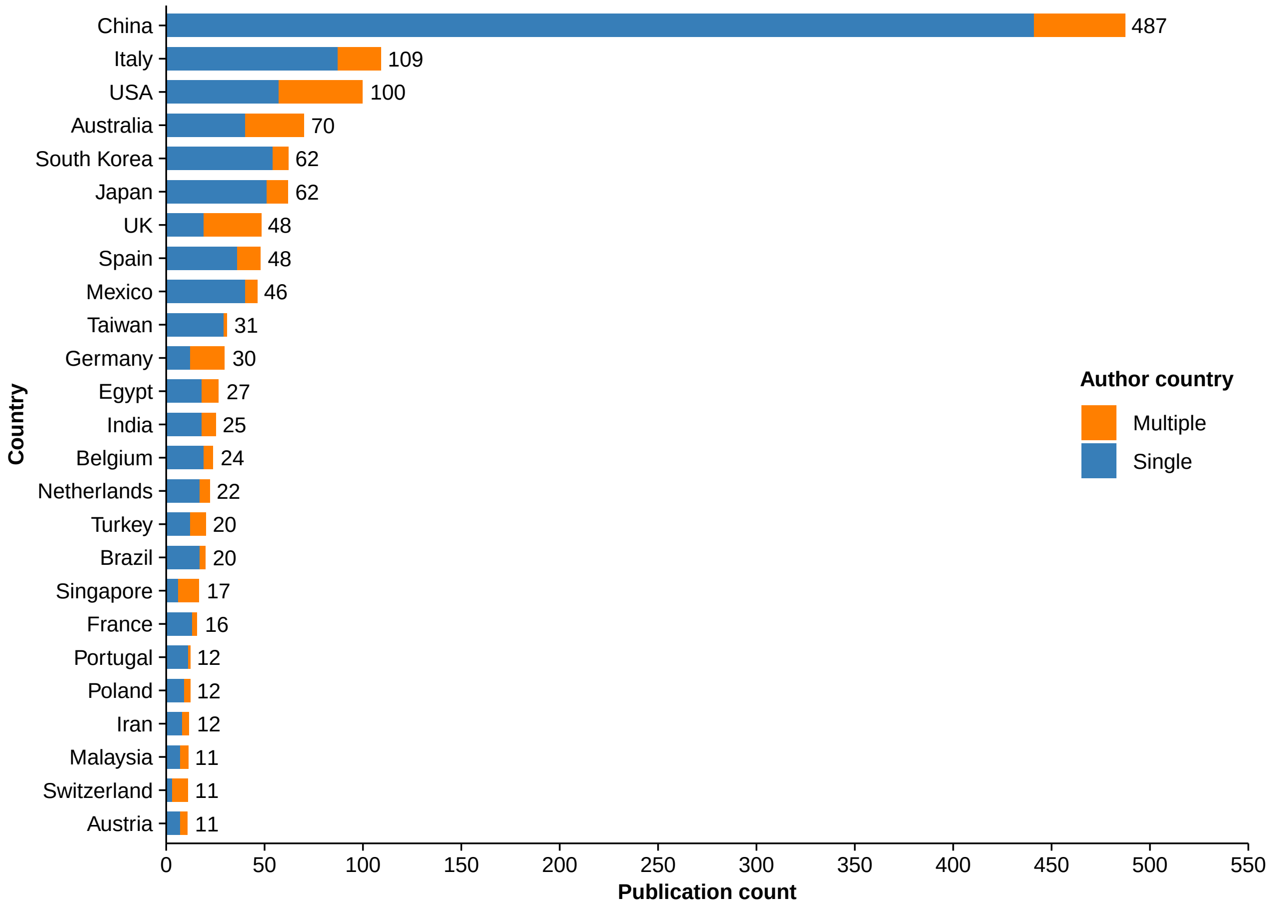

Fig. S5 Top countries producing MAFLD literature

References

1. Aria M, et al. bibliometrix : An R-tool for comprehensive science mapping analysis. Journal of Informetrics. 2017;11(4):959-75.
2. Wickham H, et al. Welcome to the Tidyverse. Journal of Open Source Software. 2019;4(43).
3. Arel-Bundock V, et al. countrycode: An R package to convert country names and country codes. Journal of Open Source Software. 2018;3(28).
4. Waltman L. A review of the literature on citation impact indicators. Journal of Informetrics. 2016;10(2):365-91.
5. Pebesma E. Simple Features for R: Standardized Support for Spatial Vector Data. The R Journal. 2018;10(1).
6. Zizka A, et al. CoordinateCleaner: Standardized cleaning of occurrence records from biological collection databases. Methods in Ecology and Evolution. 2019;10(5):744-51.
7. Csárdi G, et al., editors. The igraph software package for complex network research2006.
8. Perianes-Rodriguez A, et al. Constructing bibliometric networks: A comparison between full and fractional counting. Journal of Informetrics. 2016;10(4):1178-95.
9. Traag VA, et al. From Louvain to Leiden: guaranteeing well-connected communities. Sci Rep. 2019;9(1):5233.
10. Holten D. Hierarchical edge bundles: visualization of adjacency relations in hierarchical data. IEEE Trans Vis Comput Graph. 2006;12(5):741-8.
11. Fruchterman TMJ, et al. Graph drawing by force-directed placement. Software: Practice and Experience. 2006;21(11):1129-64.
12. Adai AT, et al. LGL: creating a map of protein function with an algorithm for visualizing very large biological networks. J Mol Biol. 2004;340(1):179-90.
